# Previous Exercise Training Has a Beneficial Effect on Renal and Cardiovascular Function in a Model of Diabetes

**DOI:** 10.1371/journal.pone.0048826

**Published:** 2012-11-07

**Authors:** Kleiton Augusto dos Santos Silva, Rafael da Silva Luiz, Rodolfo Rosseto Rampaso, Nayda Parísio de Abreu, Édson Dias Moreira, Cristiano Teixeira Mostarda, Kátia De Angelis, Vicente de Paulo Castro Teixeira, Maria Cláudia Irigoyen, Nestor Schor

**Affiliations:** 1 Nephrology Division, Department of Medicine, Federal University of Sao Paulo (UNIFESP/EPM), Sao Paulo, Brazil; 2 Hypertension Unit, Heart Institute (InCor), Medical School, University of Sao Paulo, Sao Paulo, Brazil; 3 Laboratory of Translational Physiology, Nove de Julho University, Sao Paulo, Brazil; CRCHUM-Montreal Diabetes Research Center, Canada

## Abstract

Exercise training (ET) is an important intervention for chronic diseases such as diabetes mellitus (DM). However, it is not known whether previous exercise training intervention alters the physiological and medical complications of these diseases. We investigated the effects of previous ET on the progression of renal disease and cardiovascular autonomic control in rats with streptozotocin (STZ)-induced DM. Male Wistar rats were divided into five groups. All groups were followed for 15 weeks. Trained control and trained diabetic rats underwent 10 weeks of exercise training, whereas previously trained diabetic rats underwent 14 weeks of exercise training. Renal function, proteinuria, renal sympathetic nerve activity (RSNA) and the echocardiographic parameters autonomic modulation and baroreflex sensitivity (BRS) were evaluated. In the previously trained group, the urinary albumin/creatinine ratio was reduced compared with the sedentary diabetic and trained diabetic groups (p<0.05). Additionally, RSNA was normalized in the trained diabetic and previously trained diabetic animals (p<0.05). The ejection fraction was increased in the previously trained diabetic animals compared with the diabetic and trained diabetic groups (p<0.05), and the myocardial performance index was improved in the previously trained diabetic group compared with the diabetic and trained diabetic groups (p<0.05). In addition, the previously trained rats had improved heart rate variability and BRS in the tachycardic response and bradycardic response in relation to the diabetic group (p<0.05). This study demonstrates that previous ET improves the functional damage that affects DM. Additionally, our findings suggest that the development of renal and cardiac dysfunction can be minimized by 4 weeks of ET before the induction of DM by STZ.

## Introduction

Diabetes mellitus (DM) has become an epidemic disease characterized by metabolic abnormalities and associated cardiovascular and kidney complications. A sedentary lifestyle and poor glycemic control may contribute to an increase in morbidity and mortality in DM patients and in streptozotocin (STZ)-induced DM [1], [2].

Patients with DM are at an increased risk for cardiovascular disease and often develop end-stage renal disease (ESRD). Persistent hyperglycemia may cause kidney damage, glomerular hyperfiltration, hypertension, osmotic diuresis and proteinuria [3]. Persistent hyperglycemia may also interfere with renal innervation and impair arterial baroreflex of renal sympathetic nerve activity [4], [5]. In contrast, in STZ-induced DM, renal sympathetic nerves may differentially modulate renal glucose transporters, such as GLUT1 and GLUT2 [6]. Furthermore, renal autoregulation may be impaired in this experimental model [7]. Autonomic neuropathy is present in many patients with DM and chronic kidney disease (CKD) and has been shown to decrease heart rate variability (HRV) and increase morbidity and mortality [8]. It is a relatively under-recognized predictor of adverse cardiovascular and renal outcomes in patients with nondialysis CKD [9]. In patients with CKD, impaired baroreflex sensitivity (BRS) may decrease the glomerular filtration rate (GFR) [10].

Exercise training (ET) appears to be an important intervention in protecting against chronic diseases such as DM, ESRD and heart failure [11]–[13]. ET attenuates the complications observed in diabetic animals and in humans [14]–[17]. Studies from our laboratory have demonstrated that ET has beneficial effects in spite of ongoing hyperglycemia, and animals with STZ-induced DM presented physical capacity improvement after ET [2], [14]. Several studies have evaluated autonomic responses in animals and humans undergoing a physical training program [18]–[22]. Increases in the high-frequency component of HRV have indicated the beneficial effects of ET in patients with DM [14]. In diabetic patients with kidney disease, aerobic training may improve both physical function and quality of life [11], [23].

Despite extensive evidence demonstrating the benefits of moderate exercise on the morphological and functional outcomes of several diseases, the evidence for the effects of previous ET in experimental models of DM is limited. To our knowledge, this is the first study to evaluate moderate aerobic previous ET in an experimental model of STZ-induced DM and to measure the evolution of renal and autonomic dysfunction. Thus, the aim of this study was to evaluate the effects of previous exercise training in animals with STZ-induced DM.

## Results

### Body Weight (BW), Blood Glucose and Physical Capacity

All animals started the protocols with similar BW. The final BW of the PTD group was higher than that of the D and TD groups (p<0.05). Surprisingly, the BW of the TD group did not increase over the duration of the experiment, as shown in Table 1.

**Table 1:** Body weight, blood glucose and maximal running test in control – C, sedentary diabetic – D, trained control – TC, trained diabetic – TD and previously trained diabetic – PTD groups at three different times points (initial, after induction, and final data for blood glucose; and initial, end of week 4 and final data for body weight and maximal running test).

	C	D	TC	TD	PTD
***Body weight (g)***					
**Initial**	184±2	188±2	184±2	193±2	191±3
**End of week 4**	321±9	303±5	321±9	314±5	311±7
**Final**	405±10	233±6*	379±4#	282±11*#+	343±18*#+$
***Blood glucose (mg/dL)***					
**Initial**	90±1	88±2	92±2	92±2	89±2
**After induction**	88±1	380±29*	91±1#	369±20*+	379±33*+
**Final**	87±2	497±19*&	89±3#	379±20*#+	365±19*#+
***Maximal running test (km/h)***					
**Initial**	1.10±0.09	1.10±0.07	1.10±0.07	1.20±0.07	1.20±0.06
**End of week 4**	1.20±0.06	1.20±0.08	1.20±0.06	1.20±0.07	1.90±0.10≠@
**Final**	1.20±0.10	1.00±0.05*	2.40±0.10*#	1.80±0.07*#+@	2.30±0.09*#$@

The results are expressed as the means ± SEM. ANOVA one way, * p<0.05 vs. C, # p<0.05 vs. D, + p<0.05 vs. TC, $ p<0.05 vs. TD, & p<0.05 vs. after induction, ≠ p<0.05 vs. all groups, @p<0.05 vs. initial data.

After the induction of diabetes, all of the diabetic groups had high blood glucose; however, only the TD and PTD groups demonstrated attenuation in hyperglycemia at the end of the protocol. The diabetic group (D) was hyperglycemic compared with the TD and PTD groups (p<0.05) (Table 1).

The initial physical capacity for aerobic exercise was similar for all groups. At the end of the 4^th^ week of training, the physical capacity of the PTD group had increased compared with all other groups at the same time point (p<0.05). At the end of the experimental protocol, the physical capacity of the trained groups had increased. At the end of the protocol, the physical capacity of the PTD group was higher than that of the D and TD groups (p<0.05), as shown in Table 1.

### Renal Function

**Figure 1:**
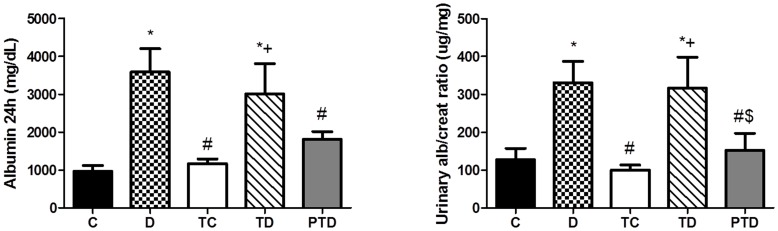
Quantitative analysis of 24 h albumin excretion (mg/dL) – A; and urinary albumin/creatinine ratio (µg/mg) – B in control – C, sedentary diabetic – D, trained control – TC, trained diabetic – TD and previously trained diabetic – PTD. The data are reported as the means ± SEM. ANOVA one way, * p<0.05 vs. C, # p<0.05 vs. D, + p<0.05 vs. TC, $ p<0.05 vs. TD.

Table 2 shows the initial and final values of renal function and protein excretion. There was no significant difference in any of the initial values between groups. The D group experienced increases in serum creatinine, proteinuria, FE_Na+_ and FE_K+_ and decreases in creatinine clearance compared with the other groups (p<0.05). The TD and PTD groups had normal creatinine clearance levels compared with the D group (p<0.05). The TD and PTD groups had lower urinary protein excretion compared with the D group (p<0.05), and the PTD group had lower protein excretion than the TD group, although this difference was not significant. With regard to albumin excretion, the PTD group had lower albumin excretion than the D group (p<0.05). Furthermore, the PTD also had a lower urine albumin/creatinine ratio compared with the D and TD groups, as shown in Figure 1. These data suggest that both trained diabetic groups were affected by exercise and that the beneficial effects of exercise were more pronounced in the PTD group.

**Table 2:** Initial and final serum creatinine, creatinine clearance, proteinuria, fractional excretion of sodium (FE_Na+_) and fraction excretion of potassium (FE_K+_) in control – C, sedentary diabetic – D, trained control – TC, trained diabetic – TD and previously trained diabetic – PTD.

	Serum Creatinine(mg/dL)		Creatinine Clearance(mL/min)		Proteinuria(mg/24 h)		FE_Na+_		FE_K+_	
	Initial	Final	Initial	Final	Initial	Final	Initial	Final	Initial	Final
**C**	0.78±0.05	0.72±0.05	1.08±0.08	1.04±0.10	15.19±2.30	17.46±1.80	0.37±0.02	0.41±0.01	3.00±0.05	3.20±0.20
**D**	0.79±0.03	1.37±0.1≠	1.03±0.07	0.26±0.07≠	15.73±1.20	90.59±5.70*@	0.44±0.02	2.40±0.30≠@	2.90±0.56	6.80±1.20≠@
**TC**	0.73±0.06	0.77±0.04	1.05±0.06	1.12±0.13	14.85±1.40	12.37±0.80#	0.36±0.02	0.41±0.07	2.90±0.20	3.10±0.34
**TD**	0.73±0.03	0.64±0.05	1.00±0.10	1.02±0.40	15.43±1.50	46.8±4.10*#+@	0.39±0.02	0.99±0.02@	3.00±0.56	3.20±0.54
**PTD**	0.73±0.05	0.70±0.05	1.18±0.09	1.19±0.16	13.42±0.70	36.89±10.20*#+	0.50±0.07	0.72±0.06	2.80±0.40	2.90±0.90

The results are expressed as the mean ± SEM. ANOVA one way, * p<0.05 vs. C, # p<0.05 vs. D, + p<0.05 vs. TC, ≠ p<0.05 vs. all groups, @p<0.05 vs. initial data.

**Figure 2:**
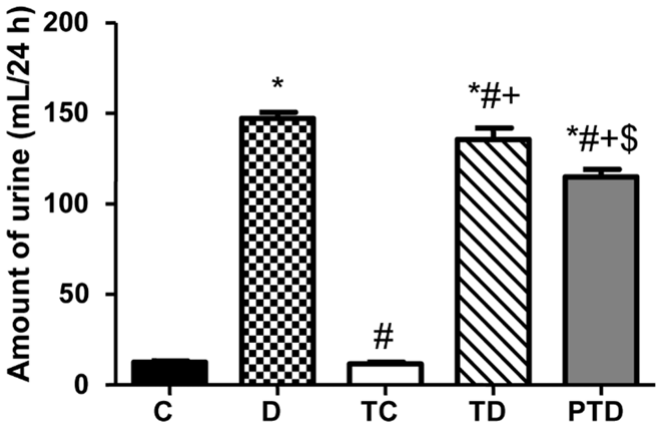
Amount of urine (mL/24 h) in control – C, sedentary diabetic – D, trained control – TC, trained diabetic – TD and previously trained diabetic – PTD. The data are reported as the means ± SEM. ANOVA one way, * p<0.05 vs. C, # p<0.05 vs. D, + p<0.05 vs. TC, $ p<0.05 vs. TD.

FE_Na+_ and FE_K+_ were lower in the TD and PTD groups compared with the D group (p<0.05). There was no significant difference between the trained diabetic groups with regard to FE_Na+_ or FE_K+_. Furthermore, the animals with DM had increases in diuresis (p<0.05). While previous ET was able to minimize the amount of urine (mL/24 h) in the TD group, the intensity of this effect was more evident in the PTD group (p<0.05), as shown in Figure 2. Proteinuria, FE_Na+_ and FE_K+_ did not significantly change during the course of the experiment in the PTD group (p>0.05). However, the D and TD groups demonstrated significant differences in these values, as shown in Table 2. These outcomes demonstrated that previous ET might delay the evolution of renal damage in STZ-induced DM.

### Echocardiography Evaluation

The echocardiography results are shown in Table 3. According to the left ventricular morphometric data, the left ventricular (LV) mass was lower in group D than in group C. Groups TC, TD and PTD had LV mass values similar to that of group C. Systolic function, expressed by fractional shortening (LVFS%), ejection fraction (LVEF%) and VCF (circ/sec) were lower in group D than in group C (p<0.05). The TD group had a higher LVEF% than group D. However, the TD group had a lower LVEF% than the C and PTD groups (p<0.05). The VCF in the D group was lower than in group C and similar that of group TD. The PTD group had a VCF similar to the C group. According to the diastolic data, the D group had a higher IVRT than groups C, TC, TD and PTD (p<0.05). Conversely, the PTD group had a lower IVRT than the TD group (p<0.05). The myocardial performance index (MPI) was higher in the D and TD groups compared with group C (p<0.05). However, the MPI of the PTD group was similar to that of the C group. Thus, both diabetic groups who underwent exercise had improved cardiac performance, but additional improvements were observed in the PTD group, as revealed in the IVRT and MPI values, indicating better adaptation.

**Table 3:** Echocardiographic parameters in control – C, sedentary diabetic – D, trained control – TC, trained diabetic – TD and previously trained diabetic – PTD.

	C	D	TC	TD	PTD
***Morphometric***					
**LV MASS(g)**	1.24±0.03	1.09±0.04*	1.3±0.05	1.19±0.04	1.22±0.07
***Systolic function***					
**LVEF(%)**	78±1.30	60±1.10*	77±1.20	65±1.50*#	72±1.10*#$
**LVFS(%)**	40±1.50	34±1.00*	41±1.10	37±0.90	40±1.60#
**VCF(circ/sec)**	0.0049±0.0003	0.0035±0.0002*	0.0048±0.0003	0.0039±0.0001*	0.0043±0.0002
***Diastolic function***					
**IVRT (ms)**	27±0.40	32±0.90*	26±0.60	29±0.80	26±0.90$
***Global index***					
**MPI**	0.32±0.020	0.45±0.050*	0.3±0.026	0.38±0.020*	0.33±0.010$

Left ventricular mass (LV mass), left ventricular ejection fraction (LVEF%), left ventricular fractional shortening (LVFS%), circumferential fiber shortening (VCF), isovolumic relaxation time (IVRT) and myocardial performance index (MPI). * p<0.05 vs. C, # p<0.05 vs. D, + p<0.05 vs. TC, $ p<0.05 vs. TD.

### Hemodynamic and Autonomic Function Evaluation

**Figure 3:**
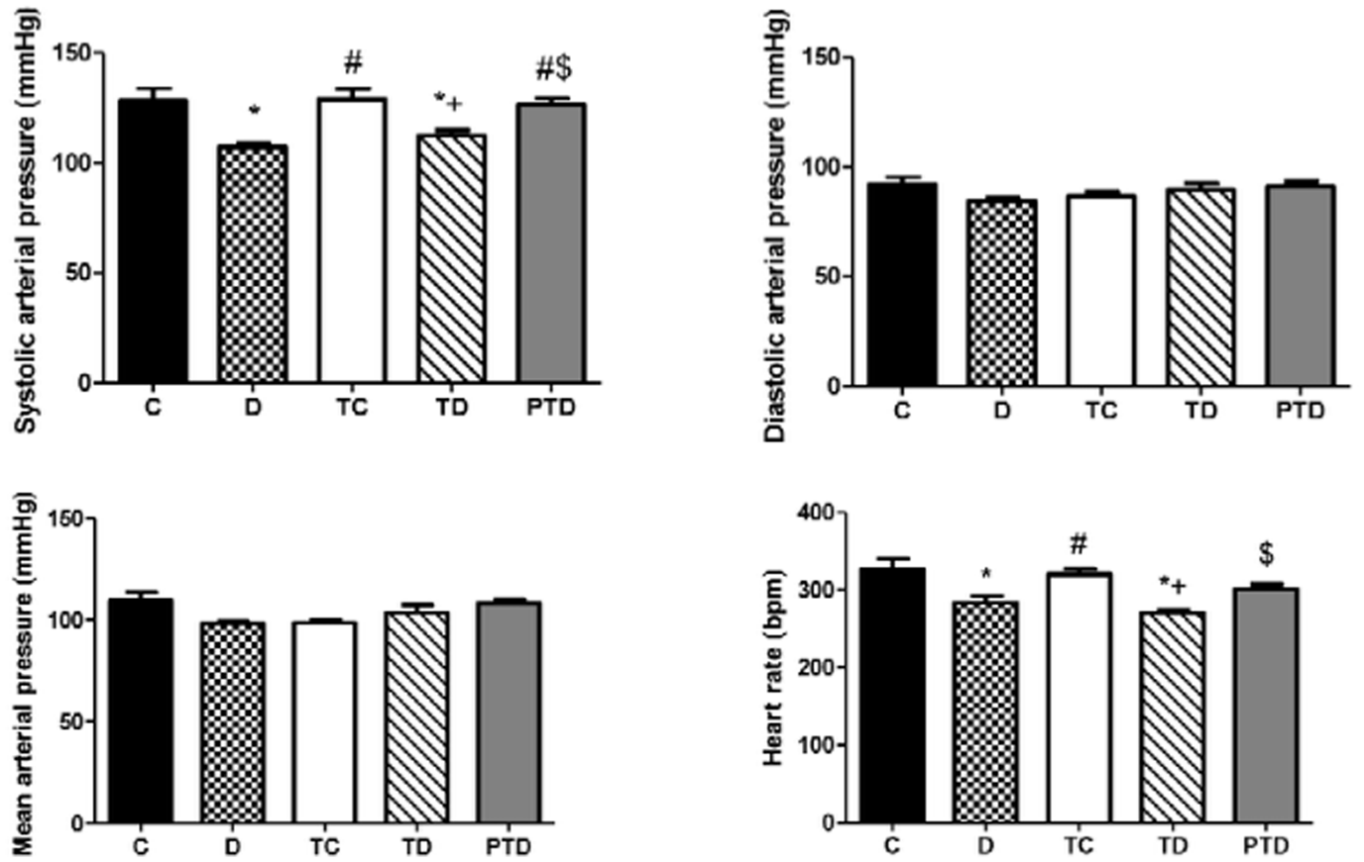
Hemodynamic parameters (systolic arterial pressure – SAP; diastolic arterial pressure – DAP; mean arterial pressure – MAP) in control – C, sedentary diabetic – D, trained control – TC, trained diabetic – TD and previously trained diabetic – PTD. The data are reported as the means ± SEM. ANOVA one way, * p<0.05 vs. C, # p<0.05 vs. D, + p<0.05 vs. TC, $ p<0.05 vs. TD.

At the end of the experimental protocol, hemodynamic parameters were assessed. The mean (MAP), systolic (SAP) and diastolic arterial pressures (DAP) along with heart rate are shown in Figure 3. The D group had a lower systolic arterial pressure and HR compared with the C and PTD groups (p<0.05). Previous ET normalized the SAP (p<0.05) and HR (p<0.05); however, the decreases in these variables did not recover in the TD group.

The results from the cardiac autonomic control measurements are summarized in Table 4. The variance in the pulse interval (PI) was lower in the diabetic groups than in any of the other groups (p<0.05). However, there was no difference between the PTD, TD and TC groups, suggesting a greater adaptation to ET in the PTD and TD groups. The RMSSD index, demonstrates vagal modulation, was higher in the TD and PTD groups than in any of the other groups (p<0.05), but ET did not affect the RMSSD index in the TC group relative to control. With regard to HRV bands, both the TD and PTD groups had a normalized LF band. However, the HF band was high in the TD group and normal in the PTD group, suggesting a better effect with regard to this parameter in the PTD group (p<0.05). By another site group D had lower HRV bands (both LF and HF), a much worse response (p<0.05). Again in this evaluation, the PTD group showed better results related to cardiac-autonomic adaptation.

**Table 4:** Cardiac autonomic control parameters in control – C, sedentary diabetic – D, trained control – TC, trained diabetic – TD and previous trained diabetic – PTD.

	PI Variance (ms^2^)	SAP Variance (mm Hg^2^)		RMSSD (ms)		LF band (ms)			HF band (ms)
**C**	95±33		24±2.90		6.15±0.50		4.2±0.86	10.71±2.30	
**D**	41±2≠		6±0.44*		5.07±0.31		1.87±0.37≠	6.46±0.59	
**TC**	107±21		31±3.00*#		7.52±0.51		4.97±1.06	15.9±2.30#	
**TD**	82±12		10±4.40*+		11.5±1.80*#+		4.92±1.38	23.62±2.70*#+	
**PTD**	93±7		12±1.70*+		11.2±0.20*#+		4.36±0.65	14.42±1.40#$	

Pulse interval variance (PI variance), Systolic arterial pressure variance (SAP variance), Root mean square of successive PI (RMSSD) index, low frequency band (LF band) and high frequency band (HF band). The results are expressed as the mean ± SEM. ANOVA one way, * p<0.05 vs. C, # p<0.05 vs. D, + p<0.05 vs. TC, $ p<0.05 vs. TD, ≠ p<0.05 vs. all groups.

**Figure 4:**
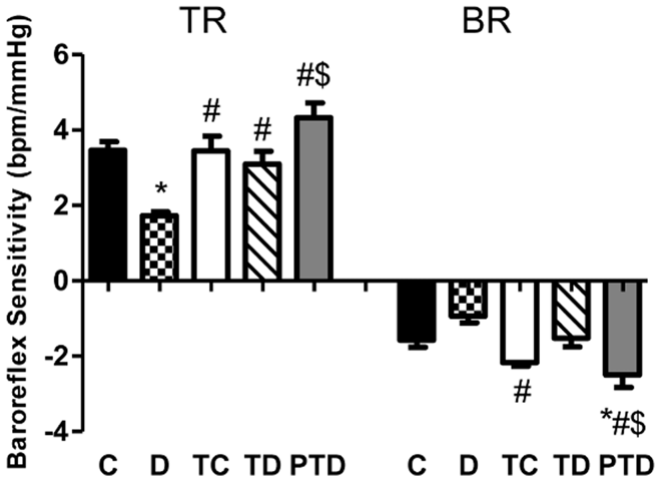
Tachycardic and bradycardic responses evaluated by vasoactive drugs (phenylephrine and sodium nitroprusside) in control – C, sedentary diabetic – D, trained control – TC, trained diabetic – TD and previous trained diabetic – PTD. The data are reported as the means ± SEM. ANOVA one way, * p<0.05 vs. C, # p<0.05 vs. D, $ p<0.05 vs. TD.

A significant decrease in baroreflex sensitivity (BRS) was observed in the D group (TR response), as shown in Figure 4. Tachycardic (TR) and bradycardic (BR) responses were lower in both the D and TD groups compared to the PTD group (p<0.05). However, only the TR parameter increased in the TD group relative to the D group (p<0.05). In the PTD group, an increase in the tachycardia and bradycardia values compared with the TD group was observed (p<0.05).

### Renal Sympathetic Nerve Activity

**Figure 5:**
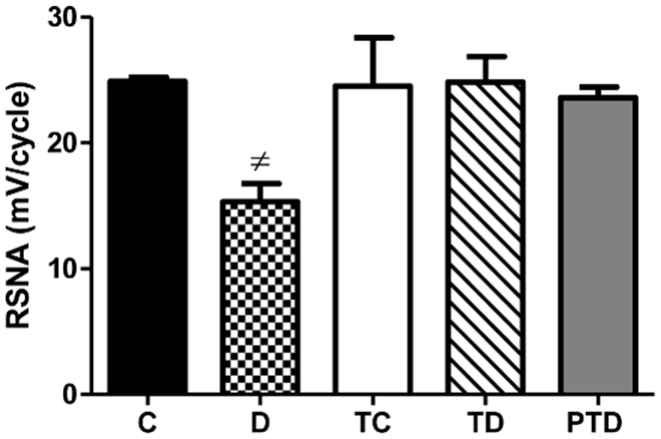
Renal sympathetic nerve activity in control – C, sedentary diabetic – D, trained control – TC, trained diabetic – TD and previous trained diabetic – PTD. The data are reported as the means ± SEM. ANOVA one way, ≠ p<0.05 vs. all groups.

The D group had lower renal nerve activity compared with any other group (p<0.05), as shown in Figure 5. ET in the diabetic groups resulted in improved RSNA compared with the D group (p<0.05). These groups demonstrated normal values with respect to the TC group.

## Discussion

The primary finding from this study was that exercise training performed prior to the induction of DM minimized the deleterious effects of DM, specifically the development of renal and cardiac autonomic dysfunction, despite hyperglycemia. These data are consistent with previous studies that showed that ET performed after the induction of DM promotes beneficial effects in STZ-induced DM. Furthermore, this is the first study, to our knowledge, to investigate the effect of aerobic ET performed 4 weeks before disease establishment.

We investigated how previous ET influenced renal function in diabetic animals by examining creatinine, proteinuria, FE_Na+_ and FE_K+_. Moreover, we analyzed RSNA and cardiac autonomic function as well as echocardiographic parameters. As expected, the diabetic animals had decreased BW and physical capacity and increased blood glucose upon the development of DM. ET attenuated hyperglycemia and weight loss and increased physical capacity, consistent with previous studies [2], [14].

Importantly, previous ET led to improvements in physical and metabolic parameters. The PTD group had over twice the physical capacity (≈2.5 fold) as that of the D group and higher physical capacity than the TD group (Table 1). These data suggest that 4 weeks of exercise training prior to the onset of DM may increase aerobic performance. In fact, exercise intolerance is present in rats with DM [14]. In 1996, el Midaoui and colleagues demonstrated that in spite of hyperglycemia, 10 weeks of aerobic ET increased the energetic capacity of skeletal muscle mitochondria in both control trained and trained diabetic animals [24]. Factors such as reduced skeletal muscle capillary numbers and GLUT4 may influence this outcome [25], [26]. Kivelä and colleagues demonstrated that expression of angiogenic growth factors and capillaries in skeletal muscle increase in controls and STZ-induced DM trained animals. These data expand on previous studies to suggest that despite previous data showing a beneficial influence of exercise in diabetic animals, additional effects are observed when exercises are performed prior to the induction of diabetes. The observed additional effects observed in the PTD versus TD are shown in Table 1 [25]. Regarding BW, the PTD group had a weight gain of approximately 8% compared with the pre-diabetes weight, as shown in Table 1. In contrast, BW decreased by approximately 24% in the D group. Studies have shown that ET can be effective in metabolic control by improving insulin sensitivity and glucose homeostasis [16], [27], [28]; our exercise protocol was able to attenuate the decrease in BW in the PTD group compared with the D and TD groups, even though both the TD and PTD groups experienced the same reduction in hyperglycemia. Moreover, the TD and PTD groups were better able to attenuate hyperglycemia compared with the D group, which may have mitigated the metabolic changes (despite the high glucose levels and absence of insulin) in these animals [29].

With regard to renal function, previous studies have shown that a single exercise session may alter renal hemodynamics due to an increase in blood flow, which activates muscles and promotes an increase in intraglomerular pressure. Therefore, an increase in efferent arteriole pressures after exercise is thought to cause an increase in hydraulic pressures and facilitate the passage of proteins throughout the glomerulus [30]–[32]. The exercise training performed in this study did not affect the urinary excretion of protein in the trained group. However, diabetic animals demonstrated impaired renal function and high excretion of protein and albumin. ET normalized and attenuated all the parameters evaluated in this study (Table 2 and Figure 1). The PTD group was able to delay the evolution of renal damage. Moreover, our results demonstrated that previously trained animals were able to attenuate the urinary albumin/creatinine ratio compared with the D and TD groups, which demonstrates a more pronounced effect of exercise training in this group. It has been suggested that ET performed after the induction of DM may ameliorate various renal function parameters such as albumin excretion [33]. STZ-induced DM animals experienced a decrease in albumin excretion after 10 weeks of aerobic ET [34]. Because the renal system is not affected by moderate aerobic ET (see Table 2), we believe that improvements in physical capacity and in the cardiovascular autonomic system might protect the renal system as a secondary adaptation. Agarwal and colleagues demonstrated that albumin excretion was significantly elevated in rats with hypertension and that 16 weeks of aerobic exercise training was able to decrease albuminuria [35]. These data suggest that both protein excretion and protein degradation may be reduced by exercise in animals with DM.

In this study, both ET and previous ET reduced proteinuria. This improvement may also be due in part to recovery of RSNA. The role of the renal nerves in the onset and progression of renal disease in DM is controversial. In 2004, Luippold and colleagues demonstrated that chronic renal denervation prevents the progression of diabetic nephropathy in early diabetes by decreasing glomerular hyperfiltration [36]. In contrast, Matsuoka and colleagues suggested that renal sympathetic denervation accelerates the development of nephropathy in an experimental diabetic model [37]. Additional studies have shown that the levels of TGF-β1 and urinary albumin were increased in an experimental diabetic model, and the authors suggested that renal denervation promoted renal injury [6], [38]. In the present study, a significant decrease in RSNA was observed in the D group, with a concomitant worsening of renal function. Moreover, exercise training performed in the TD and PTD groups was able to normalize RSNA. Taken together, these data suggest that the improvement in renal function may be linked to improvements in RSNA in the trained diabetic groups.

Researchers have observed LV systolic and diastolic dysfunction in STZ-induced DM [39], [40]. Additionally, Akula and colleagues, by echocardiographic observations, during 2, 4, 8, and 12 weeks of diabetes demonstrated that LV systolic and diastolic dysfunction were fully visible at 12 weeks [41]. Moreover, systolic and diastolic dysfunction in STZ rats were observed after 30 days by our group [42]. These animals showed reduced systolic function, as demonstrated by decreased ejection fraction and fractional shortening [42]. Furthermore, diastolic dysfunction by an increased IVRT was observed in diabetic animals compared with controls. In another study, Mihm and colleagues showed reductions in heart rate and end diastolic time after 3 days of STZ-induced DM, and these reductions progressed throughout the 56-day period. The same authors reported that systolic dysfunction was detected only after 35 days of study [43].

A possible mechanism that partially explains the cardiac dysfunction observed in these models is the decrease in calcium-sensing receptors in diabetic rats induced by STZ [44]. Studies have shown that additional autonomic dysfunction can contribute to a reduction in sarcoplasmic reticulum Ca^+^ transport ATPase (SERCA-2) expression in myocardial infarction. Additionally, severe baroreflex dysfunction by chronic sinoaortic denervation, promotes a decrease in SERCA-2 expression and increase in Na^+^-Ca^+^ exchanger expression [45]. The major finding of this study with regard to the echocardiographic parameters is that previous exercise training had a preventative effect on cardiac structure and function, induced by diabetes, an import factor favoring pre-conditioned exercise. In myocardial infarction rats, early exercise training intervention attenuates autonomic dysfunction, increases SERCA-2 expression and improves functional capacity [46].

Several studies from our laboratory and others have demonstrated impaired autonomic function in rats and humans with DM [2], [14], [47]–[50]. Our results confirm previous studies from our laboratory that demonstrate a decreased AP and HR in sedentary diabetic rats [27]. Diabetic rats that were previously exercise-trained showed increases in SAP and HR. These beneficial outcomes in SAP and HR may be explained by increases in sympathetic and parasympathetic modulation. However, the diabetic trained group did not demonstrate changes in SAP. One possible explanation is that the amount of exercise training may influence the outcome. In 2009, Mostarda and colleagues demonstrated that an improvement in SAP occurred by increasing training to two times per day [14]. Furthermore, De Angelis and colleagues demonstrated an attenuated SAP under these conditions. However, these authors did not demonstrate statistical significance in diabetic trained rats [27].

Impaired BRS and HRV in patients with CKD as well as in rats with DM and hypertension increase morbidity and mortality [10], [51]. Moreover, ET may improve hemodynamics and autonomic responses in rats with hypertension [51]. In this study, ET improves BRS and HRV in control trained and diabetic trained rats as well as in previously trained diabetic rats. Despite attenuation in BR, we did not find any significant differences in BR in diabetic trained rats compared to the D group, even with maintenance of absolute LF and HF components in both the TD and PTD groups. These differences may be explained by the degree of ET performed by the PTD group. Moreover, our laboratory demonstrated that an improvement in resting HR in diabetic trained rats and a concomitant improvement in HRV might be related to an increased BRS [14], [52].

In conclusion, previous exercise training was effective in inducing greater benefits in some parameters examined in this study. It is reasonable to suggest that these effects could also be expected in type 2 DM. Taken together, these findings indicate that previous exercise training in rats with STZ-induced DM protects against the progression of renal disease and cardiac autonomic and morphometric dysfunction.

## Methods

### Animals

All experimental procedures were conducted according to the National Institutes of Health Guidelines for the use and care of animals. The study protocol was approved by the Ethics in Research Committee of the Federal University of São Paulo (UNIFESP) (process N° 0878/08).

The experiments were performed using male Wistar rats (150 to 180 g – initial body weight) provided by the animal house of UNIFESP. The animals were housed in individual cages in a temperature-controlled room (22°C) with a 12 h dark-light cycle. The animals were randomly assigned to one of five groups: control (C, n = 6−8), trained control (TC, n = 6−8), sedentary diabetic (D, n = 6−8), trained diabetic (TD, n = 6−8) and previously trained diabetic (PTD, n = 6−8).

### Experimental Design

**Figure 6:**
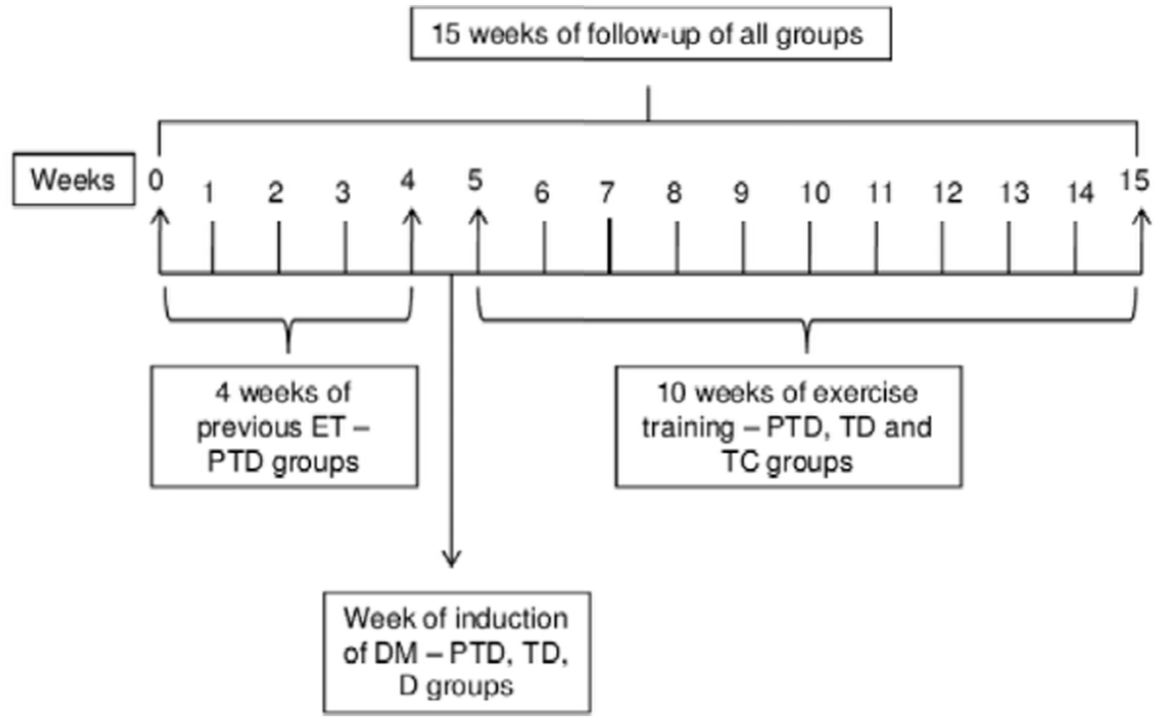
Experimental design, time course of exercise training protocol. 0– Initial week of the protocols for all groups and initiation of exercise training in the PTD group; 4– Final week of exercise training in the PTD group (4 weeks); 4 to 5– Induction of DM in the PTD, TD and D groups. During this period, the PTD group was submitted to “maintenance exercise” (≈40% maximal running test) to avoid detraining; 5– Continuation of exercise training in the PTD group and initiation of exercise training in the TD and TC groups; 15– End of the protocol and initiation of the surgical procedures.

All of the animals were followed from week 0 to week 15, as shown in Figure 6. Only the PTD group was submitted to an exercise training protocol before inducing DM, while the C, D, TC and TD groups did not exercise during before induction of DM. After this period, DM was induced in the PTD, TD and D groups. Subsequently, the PTD, TD and TC groups were submitted to an exercise training protocol for 10 weeks.

### Diabetes Mellitus Induction

DM was induced by a single injection of STZ (50 mg/kg, i.v.; Sigma Chemical Co., St. Louis, MO, USA) diluted in citrate buffer, pH 4.5, in animals weighing 300 to 320 g after 6 h of fasting.

### Maximal Running Test and Exercise Training

Functional capacity was measured using the maximal exercise test as described in the literature [53]. Briefly, all rats were submitted to a five-day adaptation period on the treadmill (Inbramed, Brazil) at a speed of 0.3 km/h for 10 minutes. After this adaptation period, we performed the maximal exercise test, which consisted of a treadmill exercise with 0.3 km/h increments every 3 minutes for each rat.

The maximal running test was performed at the beginning, at the end of the 4^th^ week and at the end of the training protocol to determine aerobic capacity and exercise training intensity. The exercise training protocol in this study consisted of an intensity of 50–70% of the maximal running test, five days per week, building up to one hour per day toward the end of the protocol. The treadmill was not inclined in this protocol. [53], [54]. After the maximal running test, the maximal physical running capacity was reached (100%). An intensity of 50–70% of the maximal running test was used. All trained groups had the same baseline level. For the PTD group (first 4 weeks), the duration of exercise was increased every week (20 min in the first week up to 1 hour at the last week for the PTD group). After this period, the TC, TD and PTD groups had the same exercise prescription and intensity. The exercise training duration was 20 min in the first week and was increased up to 1 hour by the last week. Although the PTD group underwent the same ET protocol, the physical capacity in this group was more pronounced than the TC and TD groups at the end of 4^th^ week. The minimum velocity was 0.6 km/h, and the maximum was 1.6 km/h; the running velocity was increased during the exercise training protocol. Only the TD group was not able to reach this maximum velocity during the training period, with a maximum velocity of 1.2 km/h.

### Echocardiographic Evaluation

One day after the last training session, an echocardiography was performed by an observer blinded to the treatment group, according to the guidelines of the American Society of Echocardiography. The rats were anesthetized with ketamine 80 mg/kg + xylazine 12 mg/kg, IP, and images were obtained with a 10–14 MHz linear transducer in a SEQUOIA 512 (ACUSON, Mountain View, CA, USA) to measure morphometric parameters such as left ventricular (LV) mass; systolic function evaluated by ejection fraction (LVEF%), fractional shortening (FEFS), circumferential fiber shortening (VCF) velocity and diastolic function (IVRT). A global index was quantified by the myocardial performance index (MPI). Echocardiographic parameters were measured as previously described [42], [45].

### Renal Function

For urine collection, rats were placed in metabolic cages for 24 h at the beginning and end of the protocol period (week 0–week 15). Urinary creatinine, proteinuria, sodium and potassium were analyzed. The animals (all groups) were anesthetized at the beginning and end of the protocol period (ketamine 80 mg/kg + xylazine 12 mg/kg, IP), and plasma was collected from the retro-orbital plexus (400 µL of blood) to measure serum creatinine, sodium and potassium levels. All efforts were made to minimize suffering. To evaluate renal function and proteinuria, a semi-automatic biochemical analyzer (model BIO-200F, São Paulo, São Paulo, Brazil) was used; creatinine levels were measured by the Jaffé method (Creatinina K – Colorimétrico, Picrato alcalino, Labtest Diagnóstica SA, Minas Gerais, Brazil), and proteinuria was monitored by a Sensiprot Protein Assay Kit (Labtest Diagnóstica SA, Minas Gerais, Brazil). Albumin excretion was analyzed by the Rat Albumin ELISA Quantification Set (Bethyl Laboratories, Inc. Montgomery, TX). The fractional excretion of sodium and potassium (FE_Na+_ and FE_K+_, respectively) were calculated using traditional formulas. The final samples were collected at least 24 h after the last exercise session.

### Cardiovascular Assessment

After the last urine collection, the animals were anesthetized (ketamine 80 mg/kg + xylazine 12 mg/kg, IP); all efforts were made to minimize suffering. Under anesthesia, the animals were allocated individually in a heated surgical bed and were tethered with surgical adhesive tape in a supine position. The femoral artery and vein were cannulated with catheters (PE-10) filled with 0.06 mL saline to directly measure arterial pressure and drug administration, respectively. After recovery, the rats received food and water ad libitum and were studied 48 h after catheter placement; the rats were conscious in their cages and allowed to move freely during the experiments. The arterial cannula was connected to a strain-gauge transducer (P23Db, Gould-Statham, Oxnard, CA), and AP signals were recorded using a microcomputer attached to an analog-to-digital converter board (CODAS, 2 kHz sampling frequency, Dataq Instruments, Inc., Akron, OH) [49]. HRV was determined using the standard deviation of the basal HR during the recording period. To evaluate baroreflex sensitivity (BRS), sequentially increasing bolus injections (0.1 mL) of phenylephrine (0.25 to 32 µg/kg) and sodium nitroprusside (0.05 to 1.6 µg/kg) were administered. Peak increases or decreases in mean AP after phenylephrine or sodium nitroprusside injection as well as the corresponding peak reflex changes in HR were recorded for each medication dosing. The BRS was evaluated using the mean index, thus allowing for separate analyses of bradycardic and tachycardic responses [2], [47].

### Heart Rate and Blood Pressure Variability

Time-domain analyses consisted of calculating the mean pulse interval (PI), systolic AP, PI variability and systolic AP variability as the PI variance from the respective time series. We also calculated the root mean square of successive PI (RMSSD) index differences, which is a representative index of vagal modulation. For frequency domain analyses, the entire 20 min time series of PI and systolic AP were cubic-spline interpolated (250 Hz) and decimated to be equally spaced in time. Following linear trend removal, the power spectral density was obtained by fast fourier transformation using Welch’s method over 16,384 points with a Hanning window (512) and 50% overlap. All these autonomic data were calculated throughout electrocardiographic records of AP signals, and the means were customized using MATLAB 6.0, Mathworks [55].

### Renal Sympathetic Nerve Activity

After the cardiovascular assessment, on the same day, the rats were anesthetized (pentobarbital sodium, 40 mg/kg); all efforts were made to minimize suffering. While the rats were under anesthesia, a thin bipolar platinum electrode was placed around a branch of the left renal nerve and insulated with silicone rubber (Wacker Sil-Gel 604). During the experiment, rats were kept under anesthesia (30 min), and each rat remained in the same cage used for the cardiovascular assessments (25×15×10-cm plexiglas cages with a grid floor). The signal from the nerve electrode was recorded after amplification (Tektronix 5A22N differential amplifier). The original neurogram was monitored with a storage oscilloscope (Tektronix 5111). Further processing was performed using a data-acquisition system assembled on a personal microcomputer equipped with an analog-to-digital converter (CODAS, 10 kHz sampling frequency, Dataq Instruments) [56]. The data were normalized to account for the varying intensities of the recorded signal, which is consistent with the recording instrument’s multifiber nature. Briefly, the maximal and minimal values of nerve activity (100 and 0%) were determined from the 3% of recorded cardiac cycles that showed the highest and the lowest activity levels.

### Statistical Analyses

The results are presented as the means ± SEM. The t test was used to compare the difference between initial and final data; and ANOVA (one-way) was used to compare values between groups, followed by the Student Newman-Keuls test. The significance level was established as p<0.05.
